# Characterization of work-related accidents among professionals in a public university from 2015 to 2019

**DOI:** 10.47626/1679-4435-2023-1128

**Published:** 2024-11-14

**Authors:** Marli Aparecida Reis Coimbra, Henver Ribeiro de Paiva Filho, Ana Paula Alves Araújo, Fabiana Augusta Moreira Lopes, Anderson Alves Dias, Lúcia Aparecida Ferreira

**Affiliations:** 1 Programa de Pós-Graduação em Atenção à Saúde, Universidade Federal do Triângulo Mineiro, Uberaba, MG, Brazil; 2 Programa de Pós-Graduação em Educação Física, Universidade Federal do Triângulo Mineiro, Uberaba, MG, Brazil

**Keywords:** occupational health, accidents, occupational, public health surveillance, saúde do trabalhador, acidentes de trabalho, vigilância em saúde pública

## Abstract

**Introduction:**

Accidents at work are a critical reality in public institutions and, therefore, a highlight of attention and care.

**Objectives:**

To characterize the profile of work-related accidents among professionals at a public university, from 2015 to 2019.

**Methods:**

A quantitative, retrospective, and analytical study on work-related accidents recorded by a worker’s health care sector at a public university in the interior of the state of Minas Gerais. The unit of analysis was secondary data, that is, records in Excel^®^ 2010 spreadsheets stored in the worker’s health surveillance and promotion sector from 2015 to 2019.

**Results:**

367 work accidents occurred, with a predominance of females (71.9%), the most affected professionals were those between 20 and 27 years old (26.6%), nursing (35.8%) and medical (28.9%) teams, the most prevalent were puncture wounds in the hand region (42.4%), the operating room sector (18.1%), the reactive source person for anti-HIV serology (9.3%) and workers with surface antibodies low (38.7%).

**Conclusions:**

It is noteworthy that knowledge about the profile of work accidents can favor health promotion actions in the work environment, in addition to training and continuing education for workers.

## Abstract (pt)

**Introdução:**

O acidente de trabalho é uma realidade crítica nas instituições públicas, sendo, portanto, destaque de atenção e cuidado.

**Objetivos:**

Caracterizar o perfil dos acidentes de trabalho ocorridos entre profissionais de uma universidade pública no período de 2015 a 2019.

**Métodos:**

Estudo quantitativo, retrospectivo e analítico acerca dos acidentes de trabalho registrados por um setor de atenção à saúde do trabalhador numa universidade pública no interior do estado de Minas Gerais. As unidades de análise foram dados secundários, ou seja, registros em planilhas do Excel^®^ 2010 armazenados no setor de vigilância e promoção à saúde do trabalhador no período de 2015 a 2019.

**Resultados:**

Ocorreram 367 acidentes de trabalho, com predominância do sexo feminino (71,9%). Os profissionais mais acometidos foram os de faixa etária entre 20 e 27 anos (26,6%), das equipes de enfermagem (35,8%) e médica (28,9%). O tipo mais prevalente de acidente foram as lesões perfurocortantes em região da mão (42,4%), no setor do bloco cirúrgico (18,1%), sendo a pessoa-fonte reagente para a sorologia de anti-HIV (9,3%) e trabalhadores com anticorpo contra o antígeno de superfície da hepatite B baixo (38,7%).

**Conclusões:**

Destaca-se que o conhecimento sobre o perfil dos acidentes de trabalho pode favorecer ações de promoção à saúde no ambiente laboral, além de capacitação e educação continuada aos trabalhadores.

## INTRODUCTION

The implementation of occupational health surveillance activities has been a topic of discussion since the adoption of the 1988 Constitution of the Federative Republic of Brazil.^1^ Challenges faced by occupational health reference services are complex, as understanding the health-illness process in a specific group of workers requires specialized training for the team conducting such investigations. In addition, the theoretical knowledge and training provided to professionals in this field often do not ensure the assimilation and practical application of skills in real workplace situations.^2,3^

About 2 million deaths each year are associated with exposure to occupational hazards and causes that are considered preventable.^4^ The World Health Organization (WHO) and the International Labour Organization (ILO) have developed estimates of the global burden of work-related diseases and injuries for the period 2000 to 2016. Their goal is to identify, plan, implement and evaluate policies that can prevent work-related diseases and injuries on a global scale.^4^ These organizations highlight the 2030 Sustainable Development Goals (SDGs), which aim to ensure and promote well-being and quality health for all.^4,5^

Work-related accidents (WRAs) are a serious public health problem with a direct impact on Brazil’s economy.^6^ A WRA refers to the physical or psychological harm suffered by a worker, which may result in bodily injury or functional impairment, leading to death, or loss/reduction of working capacity.^7^ In the Federal Public Administration (FPA), WRAs include those involving civil servants, nonpermanent appointees, and temporary contract workers.^7^

The work environment should be investigated through a structured epidemiological technical nexus, as it can contribute to worker illness in various dimensions: physically, through workload and pace; emotionally, through stress or fear associated with performing tasks; and cognitively, through the challenge of managing multiple instructions or maintaining high levels of concentration.^8^

The Communication on Accidents in the Public Service (CAT/SP) is a standard document used in official health examinations within the FPA to report accidents that have occurred. Based on the CAT/SP, the medical examiner establishes the causal link to the WRA, with the assistance of the Occupational Health Surveillance and Promotion Team if necessary.^7^ According to the Manual of Official Health Examinations for Federal Public Servants, every WRA must be recorded.^7^

Specialized occupational health services are responsible for promoting the health of these workers, including measures related to biosafety, reporting and monitoring of WRAs through institutional tools. These services must actively work to prevent underreporting of WRAs, provide training for those responsible for recording and following up on WRAs, and implement prevention plans.^9,10^

WRAs reduce the quality of life of workers and negatively affect their well-being, leading to changes in self-esteem^11^ and increased public health costs.^6^ Studying the profile of WRAs can help in organizing actions aimed at controlling environmental risks to workers’ health. Therefore, the objective of this study is to characterize the profile of WRAs that occurred among professionals at a public university between 2015 and 2019.

## METHODS

This is a quantitative, retrospective, and analytical study reported according to the Strengthening the Reporting of Observational Studies in Epidemiology (STROBE) guidelines.

The research analyzed secondary data on WRAs recorded between January 1, 2015, and December 31, 2019, involving professionals at a public university in the interior of the state of Minas Gerais, Brazil. This university is nationally recognized for its excellence and offers undergraduate and graduate programs as well as health services for workers. The university also has administrative and health areas, including a clinical hospital that serves as a reference for highly complex care within the Brazilian Unified Health System (SUS).

Data were provided in Microsoft Office Excel spreadsheets by the surveillance and health promotion sector of the Department of Health Care for Public Servants (Departamento de Atenção à Saúde do Servidor, DASS), which is part of the Pro-Rectory of Human Resources (Pró-Reitoria de Recursos Humanos, PRORH) and is linked to the Integrated Subsystem for Health Care for Federal Public Servants (Subsistema Integrado de Atenção à Saúde do Servidor Público Federal, SIASS). DASS investigates WRAs involving individuals in temporary positions, volunteers, public servants hired through competitive examinations or appointed to permanent positions as well as public servants reassigned or seconded to work at the university.

During the study period, the university employed 2,098 public servants, along with 50 volunteers and 220 medical and nonmedical residents, for a total of 2,368 workers. The researchers developed a data collection instrument to extract the following information from the WRA records of these workers: gender (female, male) and age group (20-27 years, 28-33 years, 34-39 years, 40-45 years, 46-51 years, 52-57 years, 58-63 years, and ≥ 64 years). The age groups were categorized according to a study that assessed the sociodemographic and occupational profile of workers who experienced WRAs.^12^ Data collected from WRAs included work shift (morning, afternoon, night), season (1st quarter, 2nd quarter, 3rd quarter, 4th quarter), sector in which the accident occurred, occupation of the injured workers, and type of WRA. Clinical data were also collected, including immunization card status (complete, incomplete, no card), positive serology of the source person, antibodies to HIV, hepatitis C virus (anti-HCV), hepatitis B surface antigen (HBsAg), and surface antibodies (anti-HBs). Anti-HBs levels were categorized as < 10 (no immunity to hepatitis B) and ≥ 10 (considered desirable for immunity to hepatitis B) based on the institution’s laboratory reference values.

Tables provided contained missing data on age, industry, occupation, type of work-related accident (WRA), and clinical information. However, the profile of WRAs could still be evaluated. Data collected were managed by using Microsoft Office Excel^®^ 2010 and analyzed by using the Statistical Package for the Social Sciences (SPSS) software, version 21.0. Descriptive statistics were performed by using absolute and relative frequencies.

The study was approved by the research ethics committee (REC) at the participating institution (approval number 4.182.445). Professionals were assigned numbers in the records to prevent their identification. Consequently, the required informed consent (RIC) was waived by the REC.

## RESULTS

A total of 367 WRAs were recorded between 2015 and 2019, with the highest prevalence occurring in 2016 (3.42%). There was a predominance of WRAs among female workers (71.9%) and among workers aged 20 to 27 years (26.6%), which was the most affected age group in all recorded years (Table 1).

**Table 1:** Characterization of work-related accidents among professionals at a public university from 2015 to 2019, by sociodemographic variables

Variables	2015	2016	2017	2018	2019	Total
	n (%)	n (%)	n (%)	n (%)	n (%)	n (%)
Sex						
Female	54 (70.1)	61 (75.3)	56 (75.7)	58 (75.3)	35 (60.3)	264 (71.9)
Male	23 (29.9)	20 (24.7)	18 (24.3)	18 (24.7)	23 (39.7)	103 (28.1)
Age group^*^ (years)						
20-27	15 (19.5)	19 (23.8)	21 (28.4)	27 (35.5)	15 (25.9)	97 (26.6)
28-33	15 (19.5)	14 (17.5)	10 (13.5)	18 (23.7)	5 (8.6)	62 (17.0)
34-39	12 (15.6)	12 (15.0)	17 (23.0)	9 (11.8)	13 (22.4)	63 (17.3)
40-45	9 (11.7)	7 (8.8)	3 (4.1)	10 (13.2)	7 (12.1)	36 (9.9)
46-51	4 (5.2)	8 (10.0)	3 (4.1)	2 (2.6)	6 (10.3)	23 (6.3)
52-57	11 (14.3)	14 (17.5)	13 (17.6)	5 (6.6)	4 (6.9)	47 (12.9)
58-63	6 (7.8)	2 (2.5)	7 (9.5)	5 (6.6)	6 (10.3)	26 (7.1)
≥ 64	5 (6.5)	4 (5.0)	0 (0.0)	0 (0.0)	2 (3.4)	11 (3.0)

Source: Compiled from the records of the Department of Health Care for Public Servants (Departamento de Atenção à Saúde do Servidor, DASS), 2022.

* Missing data.

WRAs occurred most frequently during the morning shift (46.4%), in the second quarter of each year (31.6%), and within hospital sectors, with the surgical block (18.1%) and adult emergency department (12.9%) being the most prominent. Nonhospital sectors accounted for 19.4% of the WRAs recorded during the period (Table 2).

**Table 2:** Characterization of work-related accidents among professionals at a public university from 2015 to 2019, by work variables

Variables	n	%
Work shift		
Morning	166	46.4
Afternoon	119	33.2
Night	73	20.4
Time of year (quarter)		
1st	69	18.8
2nd	116	31.6
3rd	104	28.3
4th	78	21.3
Sectors^*^		
Surgical block	42	18.1
Adult emergency department	30	12.9
Orthopedics	13	5.6
Renal transplant unit	11	4.7
Gynecology and obstetrics	9	3.9
Medical clinic	8	3.4
Coronary intensive care	6	2.6
Radiology/Imaging service	6	2.6
Adult intensive care	6	2.6
Outpatient clinic	6	2.6
Laboratory	5	2.2
Neonatal intensive care	5	2.2
Infectious diseases	5	2.2
Pediatrics	5	2.2
Surgical clinic	4	1.7
Sterilized materials center	4	1.7
Others	10	4.3
Administrative	45	19.4

Source: Compiled from the records of the Department of Health Care for Public Servants (Departamento de Atenção à Saúde do Servidor, DASS), 2022.

* Missing data.

The positions most affected by work-related accidents (WRAs) were those in the nursing team, which includes nurses and nursing technicians (35.8%), followed by the medical team, which includes physicians and medical residents (28.9%) (Table 3).

**Table 3:** Characterization of work-related accidents among professionals at a public university from 2015 to 2019, by occupation

Occupation^*^	2015	2016	2017	2018	2019	Total
	n (%)	n (%)	n (%)	n (%)	n (%)	n (%)
Nursing team	29 (37.7)	30 (38.0)	32 (43.2)	29 (38.2)	13 (22.8)	130 (35.8)
Medical team	28 (36.4)	19 (24.1)	13 (17.6)	24 (31.6)	21 (36.8)	105 (28.9)
Volunteer	1 (1.3)	11 (13.9)	14 (18.9)	16 (21.1)	4 (7.0)	46 (12.7)
Administrator/Assistant	5 (6.5)	5 (6.5)	2 (2.7)	2 (2.6)	1 (1.8)	15 (4.1)
Accountant/Lawyer	0 (0.0)	4 (5.1)	5 (6.8)	1 (1.3)	3 (5.3)	13 (3.6)
Engineer	0 (0.0)	2 (2.5)	1 (1.4)	1 (1.3)	3 (5.3)	7 (1.9)
Information technology technician	0 (0.0)	1 (1.3)	1 (1.4)	1 (1.3)	4 (7.00	7 (1.9)
Radiology technician	3 (3.9)	1 (1.3)	0 (0.0)	1 (1.3)	0 (0.0)	5 (1.4)
Electrician/Electrical technician	2 (2.6)	0 (0.0)	1 (1.4)	0 (0.0)	1 (1.8)	4 (1.1)
Multiprofessional resident	4 (5.2)	0 (0.0)	0 (0.0)	0 (0.0)	0 (0.0)	4 (1.1)
Psychologist	0 (0.0)	0 (0.0)	2 (2.7)	0 (0.0)	2 (3.5)	4 (1.1)
Surgical instrument technician	3 (3.9)	0 (0.0)	0 (0.0)	0 (0.0)	0 (0.0)	3 (0.8)
Laboratory technician	3 (3.9)	0 (0.0)	0 (0.0)	0 (0.0)	0 (0.0)	3 (0.8)
Support/Maintenance services	0 (0.0)	1 (1.3)	1 (1.4)	0 (0.0)	0 (0.0)	2 (0.6)
Pharmacist/Pharmacy technician/Chemist	2 (2.6)	0 (0.0)	0 (0.0)	0 (0.0)	0 (0.0)	2 (0.6)
Dentist	0 (0.0)	0 (0.0)	0 (0.0)	1 (1.3)	0 (0.0)	1 (0.3)
Construction worker	0 (0.0)	1 (1.3)	0 (0.0)	0 (0.0)	0 (0.0)	1 (0.3)
Physical educator	0 (0.0)	1 (1.3)	0 (0.0)	0 (0.0)	0 (0.0)	1 (0.3)
Physical therapist	0 (0.0)	0 (0.0)	1 (1.4)	0 (0.0)	0 (0.0)	1 (0.3)
Other areas	0 (0.0)	3 (3.9)	1 (1.4)	0 (0.0)	5 (8.8)	9 (2.6)

Source: Compiled from the records of the Department of Health Care for Public Servants (Departamento de Atenção à Saúde do Servidor, DASS), 2022.

* Missing data.

The most common types of WRAs were biological, with sharps injuries to the hand being the most common (42.4%), followed by falls/contusions (17.2%). WRAs involving biological material in the ocular conjunctiva and during transit accounted for 12.4% and 10.1%, respectively (Table 4).

**Table 4:** Characterization of work-related accidents among professionals at a public university from 2015 to 2019, by type of accident

Type of accident	2015	2016	2017	2018	2019	Total
	n (%)	n (%)	n (%)	n (%)	n (%)	n (%)
Sharps injury to the hand	36 (46.8)	35 (44.3)	34 (46.6)	19 (24.7)	19 (32.8)	143 (42.4)
Fall/contusion	9 (11.7)	17 (21.5)	18 (24.6)	10 (13.0)	4 (6.8)	58 (17.2)
Biological material in ocular conjunctiva	5 (6.5)	5 (6.4)	3 (4.1)	13 (16.9)	16 (27.6)	42 (12.4)
Transit accident	8 (10.4)	10 (12.7)	5 (6.8)	8 (10.4)	3 (5.2)	34 (10.1)
Biological material on intact skin^*^	3 (3.9)	3 (3.8)	2 (2.7)	2 (2.6)	2 (3.4)	12 (3.5)
Chemical substance exposure	4 (5.2)	3 (3.8)	1 (1.4)	1 (1.3)	1 (1.7)	10 (2.9)
Biological material on non-intact skin	2 (2.6)	0 (0.0)	0 (0.0)	4 (5.2)	1 (1.7)	7 (2.1)
Sharps injury without biological material	1 (1.3)	2 (2.5)	3 (4.1)	0 (0.0)	0 (0.0)	6 (1.8)
Burn	1 (1.3)	1 (1.3)	0 (0.0)	2 (2.6)	1 (1.7)	5 (1.4)
Accident with surgical instruments	1 (1.3)	1 (1.3)	1 (1.4)	1 (1.3)	1 (1.7)	5 (1.4)
Exposure to physical agent	3 (3.9)	0 (0.0)	0 (0.0)	0 (0.0)	0 (0.0)	3 (0.9)
Physical assault	0 (0.0)	0 (0.0)	2 (2.7)	0 (0.0)	1 (1.7)	3 (0.9)
Crushing of limb and/or body part	1 (1.3)	0 (0.0)	0 (0.0)	1 (1.3)	0 (0.0)	2 (0.6)
Limb fracture	0 (0.0)	0 (0.0)	0 (0.0)	1 (1.3)	1 (1.7)	2 (0.6)
Needle recapping^*^	0 (0.0)	0 (0.0)	0 (0.0)	1 (1.3)	0 (0.0)	1 (0.3)

Source: Compiled from the records of the Department of Health Care for Public Servants (Departamento de Atenção à Saúde do Servidor, DASS), 2022.

* Missing data.

Of all workers, 4.4% had incomplete vaccination records and 38.7% had anti-HBs levels below the laboratory reference value. The source person tested positive for anti-HIV in 9.3% of cases, for anti-HCV in 3.5%, and for HBsAg in 1.9% (Table 5).

**Table 5:** Characterization of work-related accidents among workers at a public university from 2015 to 2019, by clinical variables

Variables	n	%
Vaccination card^*^		
Complete	103	26.1
Incomplete	16	4.4
No vaccination card	3	0.8
Source person with positive serology^*^		
HIV	34	9.3
Hepatitis C	13	3.5
Hepatitis B	7	1.9
Anti-HBs†		
< 10	142	38.7
≥ 10	165	45.0

Source: Compiled from the records of the Department of Health Care for Public Servants (Departamento de Atenção à Saúde do Servidor, DASS), 2022.

* Missing data.

† Reference values from the hospital laboratory: < 10 indicates no antibodies for hepatitis B; ≥ 10 indicates a desirable level of immunity for hepatitis B.

## DISCUSSION

The WRAs that occurred during the observation period had an average prevalence of 3.10% over the years, with a predominance of women, who accounted for almost two-thirds of the sample. This finding is consistent with previous studies and can be explained by the fact that many occupations, particularly in the health sector, are predominantly held by women.^13^

In this study, WRAs were more prevalent among younger workers, particularly those between the ages of 20 and 27. Similar findings were observed in another study using a comparable age group.^14^ In a separate study of accidents involving exposure to biological materials in Brazil over a seven-year period, the most affected age group was between 25 and 29 years.^13^

The higher prevalence of WRAs among younger workers has been documented in the literature, suggesting that these incidents may be related to inexperience and lack of technical knowledge, possibly due to educational limitations.^15^ In addition, this study was conducted at a public university that includes a teaching hospital, where incidents involving residents may have contributed to the higher prevalence among younger workers. This finding is consistent with a study conducted in Türkiye among emergency medicine residents who reported experiencing at least one WRA in the previous 12 months.^16^

In the present study, the surgical block was the area with the highest number of recorded WRAs. Similar results have been reported in a study conducted at a reference hospital for cancer treatment in Brazil^17^ as well as at a study conducted in an Italian hospital, where the surgical unit was identified as the area with the highest incidence of biological accidents.^18^ This can be attributed to the specific nature of the surgical area, where more invasive procedures are performed.

The occurrence of WRAs was more pronounced in the morning shift and in the hospital sector compared to other sectors, similar to the findings of another study.^19^ The hospital involved in this study tends to concentrate most of its activities in the morning due to the availability of professionals and scheduled procedures.

Dentists had the lowest prevalence of WRAs during the study period; however, they represent a smaller workforce in the facility, resulting in fewer procedures. Conversely, the health care workers who make up the largest operational workforce-the nursing and medical teams-were the most affected. Nursing involves 24-hour patient care that requires close physical contact, invasive procedures, and the use of sharps.^20^ As a result, these workers are exposed to biological materials on a daily basis, increasing their risk of WRAs. In addition, the workload of these workers, which often includes double shifts without rest periods, exacerbates this risk. In this context, in-service education and periodic training on biological risk awareness may be lacking.^20^

An Italian study concluded that nursing is one of the professions most affected by biological WRAs.^21^ Similar findings regarding the incidence of WRAs among nurses were reported in a study conducted in Egypt.^22^ Nurses make up the largest segment of the health care workforce and are involved in procedures that expose them to morbidity and absenteeism, creating a higher risk for WRAs.^14^

Physicians and medical residents are also frequently exposed to WRAs. This is due to the wide range of procedures they perform, from simple tasks such as suturing to more complex ones such as orotracheal intubation, venous dissection, and catheter and drain insertion.^23^ These procedures are often performed when patients are in life-threatening situations, which increases the likelihood of accidents during emergency care.^23^ In addition, residents are young professionals who are still gaining skills and experience, making them more vulnerable to accidents.^24^

Because of the occupational dynamics of WRAs involving biological exposure, the prevalence of hand injuries in this study is consistent with the literature. Another study conducted in Brazil found similar results for percutaneous injuries involving organic material.^25^ In addition, a study conducted at a hospital in Italy showed that percutaneous accidents involving blood were more common during puncture procedures.^18^ These WRAs are closely related to the nature of the tasks performed, particularly by health care workers.

The biological occupational risks faced by these categories of health care workers underscore the importance of immunization. In this study, nearly 5% of participants had incomplete vaccination records and approximately 40% had anti-HBs levels below the laboratory reference value. This finding highlights the susceptibility of these workers to exposure to hepatitis B virus (HBV) in the event of a biological occupational injury. In addition, the source individual tested positive for HIV, hepatitis C, and hepatitis B. HBV is highly resistant in the environment and can survive for up to a week in dried blood on external surfaces.^26^

Another study conducted in the state of Amapá showed that more than 80% of professionals were vaccinated against HBV.^25^ Similarly, a study in Italy reported that although many professionals were vaccinated, some of them had not completed the full vaccination schedule.^18^

A study conducted in Ethiopia showed lower vaccination rates among health care workers.^27^ In contrast, China showed good HBV vaccination coverage, although there is still a need to establish a national vaccination policy.^28^ These findings suggest that in some countries, HBV vaccination coverage among health care workers remains very low, particularly in countries with lower levels of economic development.

Strategies to encourage vaccination can be promoted by creating opportunities for dialogue about workplace exposures.^29^ Therefore, there is a need to implement institutional vaccination campaigns and regularly monitor immunization records to ensure that workers are adhering to the available vaccines.

In a study conducted in India, the rates of source persons testing positive for anti-HIV, anti-HCV, and anti-HBsAg were higher^30^ than those found in the present study. These findings suggest that regardless of the number of source persons with positive serologies, there is a need to implement protective measures and training programs to reduce WRAs.

Failure to use proper protective measures or safety equipment, combined with workload and overconfidence, can contribute to WRAs. The risk and susceptibility to WRAs in professional practice are related to individual, cultural, biological, collective, and structural factors in the work environment. Therefore, strategies for organizing and controlling WRAs are related to political, organizational, and individual behavioral factors in response to risk.^31^

Public health policies need to be developed and implemented to ensure decent working conditions and job satisfaction. In addition, coordination of education and training is essential to broaden the discussion on occupational risks, WRAs, and vulnerability in health care practices, thereby influencing workplace policies and overall well-being.^31^

This study is limited by the use of previously recorded spreadsheets that contained some incomplete information, preventing a more comprehensive analysis. The data in the spreadsheets were limited, lacking health-related details or information on the effects of the WRAs. A similar problem of incomplete data in reporting forms was reported in another study conducted in Brazil.^19^

## CONCLUSIONS

This study identified that female workers, younger individuals, the nursing and medical teams, sharps injuries to the hand, the surgical block sector, source persons testing positive for anti-HIV, and low anti-HBs levels were the primary findings related to WRAs.

These results highlight the need for increased attention to professionals exposed to biological risks and for monitoring of hepatitis B vaccination. Understanding the profile of WRAs can help implement technical adaptations that benefit both the individual and the workplace.

## References

[1] Brasil, Presidência da República (1988). Constituição da República Federativa do Brasil de
1988.

[2] Jackson Filho JM, Garcia EG, David HG, Duracenko SRC, Simonelli AP. (2019). Acidentes de trabalho e atuação do Centro de
Referência em Saúde do Trabalhador nas páginas do
Jornal de Piracicaba entre 2007 e 2014. Interface (Botucatu).

[3] Vianna LCR, Ferreira AP, Vasconcellos LCF, Bonfatti RJ, Oliveira MHB. (2017). Vigilância em Saúde do Trabalhador: um estudo
à luz da Portaria nº 3.120/98. Saude Debate.

[4] World Health Organization, International Labour
Organization (2021). WHO/ILO joint estimates of the work-related burden of disease and
injury, 2000-2016: global monitoring report.

[5] Organização das Nações Unidas (2022). Objetivos de Desenvolvimento Sustentável.

[6] Organização Internacional do Trabalho,
Observatório de Segurança e Saúde no
Trabalho (2021). Série SmartLab de trabalho decente: gastos com doenças e
acidentes do trabalho chegam a R$ 100 bi desde 2012.

[7] Brasil, Ministério do Planejamento, Desenvolvimento e
Gestão (2017). Manual de perícia oficial em saúde do servidor
público federal.

[8] Cardoso ACM. (2015). O trabalho como determinante do processo
saúde-doença. Tempo Soc.

[9] Silva AID, Machado JMH, Santos EGOB, Marziale MHP. (2011). Acidentes com material biológico relacionados ao trabalho:
análise de uma abordagem institucional. Rev Bras Saude Ocup.

[10] Souza HP, Otero UB, Silva VSP. (2019). Perfil dos trabalhadores de saúde com registros de
acidentes com material biológico no Brasil entre 2011 e 2015:
aspectos para vigilância. Rev Bras Med Trab.

[11] Santos SVM, Macedo FRM, Silva LA, Resck ZMR, Nogueira DA, Terra FS. (2017). Work accidents and self-esteem of nursing professional in
hospital settings. Rev Latino-Am Enferm.

[12] Bertelli C, Martins BR, Krug SBF, Petry AR, Fagundes PS. (2020). Acidentes de trabalho com material biológico: perfil
sociodemográfico e ocupacional dos trabalhadores
afetados. Rev Bras Med Trab.

[13] Miranda FMD, Cruz EDA, Félix JCV, Kalinke LP, Mantovani MF, Sarquis LMM. (2017). Profile of Brazilian workers victims of occupational accidents
with biological fluids. Rev Bras Enferm.

[14] Maraschin MS, Tauffer J, Berticelli M, Carmello SKM, Kupka FS. (2018). Acidentes de trabalho com exposição à
material biológico notificados em um hospital de
ensino. Var Sci - Ci Saude.

[15] Gomes SCS, Caldas AJM. (2019). Incidência de acidentes de trabalho com
exposição a material biológico em profissionais de
saúde no Brasil, 2010-2016. Rev Bras Med Trab.

[16] Yalcin Ocak N, Yesilaras M, Eyler Y, Hakoglu O. (2022). Occupational accidents of emergency medicine residents in
Turkey. Int J Occup Saf Ergon.

[17] Quixabeiro EL, Hennington ÉA. (2021). Occupational exposure to sharp instrument injuries in a federal
hospital. Rev Bras Med Trab.

[18] Stefanati A, Brosio F, Kuhdari P, Baccello V, De Paris P, Nardini M (2017). [Incidence of biological accidents at work and immune status for
vaccine-preventable diseases among resident physicians in specialist
training at Ferrara University Hospital]. Ig Sanita Pubbl.

[19] Alvares JK, Pinheiro TMM, Santos AF, Oliveira GL. (2015). Avaliação da completitude das
notificações compulsórias relacionadas ao trabalho
registradas por município polo industrial no Brasil, 2007 -
2011. Rev Bras Epidemiol.

[20] Vieira KMR, Vieira Júnior FU, Bittencourt ZZLC. (2020). Subnotificação de acidentes de trabalho com
material biológico de técnicos de enfermagem em hospital
universitário. Rev Baiana Enferm.

[21] Vitale E, Guglielmi V, Iosca M, Celani F. (2021). [Biological injuries in a hospital in Puglia: an observational
study between nurses and nursing students]. G Ital Med Lav Ergon.

[22] Sabaa MA, Hassan AM, Abd-Alla AK, Hegazy EE, Amer WH. (2022). Needle-stick and sharps injuries: awareness, prevalence and risk
factors of a global problem in healthcare workers at Tanta University
Hospitals, Egypt. Int J Occup Saf Ergon.

[23] Souza RT, Bica CG, Mondadori CS, Ranzi AD. (2012). Avaliação de acidentes de trabalho com materiais
biológicos em médicos residentes, acadêmicos e
estagiários de um hospital-escola de Porto Alegre. Rev Bras Educ Med.

[24] La-Rotta EIG, Garcia CS, Pertuz CM, Miquilin IOC, Camisão AR, Trevisan DD (2020). Conhecimento e adesão como fatores associados a acidentes
com agulhas contaminadas com material biológico: Brasil e
Colômbia. Cienc Saude Colet.

[25] Santos Júnior GT, Araújo MHM, Araújo GS, Santos BEF, Costa Junior RR. (2021). Characterization of occupational accidents occurred at the
Occupational Health Referral Center of the state of Amapá between
2007 and 2016. Rev Bras Med Trab.

[26] Santos LT, Rocha FLR, Marziale MHP. (2018). Agulhas com dispositivos de segurança e a
prevenção de acidentes: revisão
integrativa. Rev Bras Enferm.

[27] Abebaw TA, Aderaw Z, Gebremichael B. (2017). Hepatitis B virus vaccination status and associated factors among
health care workers in Shashemene Zonal Town, Shashemene, Ethiopia: a cross
sectional study. BMC Res Notes.

[28] Liu Y, Ma C, Jia H, Xu E, Zhou Y, Zhang Z (2018). Knowledge, attitudes, and practices regarding hepatitis B
vaccination among hospital-based doctors and nurses in China: results of a
multi-site survey. Vaccine.

[29] Souza FO, Araújo TM. (2018). Exposição ocupacional e vacinação
para hepatite B entre trabalhadores da atenção primária
e média complexidade. Rev Bras Med Trab.

[30] Sharma R, Gupta P, Jelly P. (2020). Pattern and serological profile of healthcare workers with
needle-stick and sharp injuries: a retrospective analysis. J Family Med Prim Care.

[31] Santos JLG, Vieira M, Assuiti LFC, Gomes D, Meirelles BHS, Santos SMA. (2012). Risco e vulnerabilidade nas práticas dos profissionais de
saúde. Rev Gaucha Enferm.

